# Partially anaortic clampless off-pump coronary artery bypass prevents neurologic injury compared to on-pump coronary surgery: a propensity score-matched study on 286 patients

**DOI:** 10.1007/s00380-015-0748-z

**Published:** 2015-10-23

**Authors:** Carlo Bassano, Emanuele Bovio, Floriano Uva, Simona Iacobelli, Nicola Iasevoli, Andrea Farinaccio, Giovanni Ruvolo

**Affiliations:** 1 Operative Units of Cardiac Surgery, Tor Vergata University, Rome, Italy; 2 Operative Units of Anesthesiology, Tor Vergata University, Rome, Italy; 3 Interdepartmental Center of Biostatistics and Bioinformatics, Tor Vergata University, Rome, Italy

**Keywords:** Off-pump surgery, CABG, Neurologic injury, Clampless, Stroke

## Abstract

Anaortic coronary artery bypass proved to prevent early neurologic injury compared to on-pump CABG. The Cardica PAS-Port^®^ is a fully automated device that might be able to perform proximal aorto-venous anastomoses without an increased embolic risk. We evaluated early post-operative neurologic outcome in a matched population following clampless OPCAB (CCAB: either “all-arterial” or with automatically anastomosed venous grafts) or on-pump CABG. 366 consecutive patients were submitted to isolated coronary bypass by a single surgeon experienced in both off and on-pump procedures between January 2009 and December 2013. Of these patients, 223 underwent a clampless off-pump revascularization. After propensity score matching, 143 pairs were selected, who received either off-pump or on-pump surgery. In the off-pump group, CCAB was performed with an all-arterial approach (*n* = 33) or with automated proximal anastomosis of the venous graft(s) by means of the Cardica PAS-Port^®^ connector (*n* = 110). Neurologic injury was defined as non-reversible (NRNI: lethal coma or stroke) or reversible (RNI: TIA or delirium). Operative mortality was 2.4 % (CCAB 1.4 %; CABG 3.5 %; *p* = 0.14). The global rate of early neurologic injury was 5.6 % (CCAB 2.1 vs. CABG 9.1 %; *p* = 0.006). Incidence was 1.4 % for NRNI (CCAB 0 vs. CABG 2.8 %; *p* = 0.04) and 4.2 % for RNI (CCAB 2.1 vs. CABG 6.3 %; *p* = 0.06). No differences were found among other major perioperative outcomes. CCAB prevents both early post-operative RNI and NRNI. This result can be achieved with a totally anaortic strategy and also with the aid of a fully automated device for proximal aorto-venous anastomoses.

## Introduction

It is not still clear whether OPCAB is actually superior to CABG in preventing post-operative neurologic damage [1, 2]. Neurologic injury seems to be mostly related to atheroembolism from the ascending aorta and to clamping strategies [3, 4]. Therefore, the anaortic technique should be effectively superior to any other strategy in reducing the incidence of stroke by avoiding any aortic manipulation [5–8]. Indeed, the anaortic coronary surgery is more often based on the exclusive use of either both internal thoracic arteries (ITA) in different configurations, or by adding other conduits (radial artery or saphenous vein) anastomosed proximally to the ITA grafts. This approach includes a possibly increased risk of sternal wound dehiscence and a restricted number of flow sources to the coronary bed [9]. Facilitating devices for proximal venous graft anastomoses help avoiding side-clamping of the aorta but can be the cause of microembolic phenomena [10]. The Cardica PAS-Port^®^ device (Cardica Inc., Redwood City, CA, USA) allows to achieve proximal aorto-venous automated anastomoses with potential reduction of micro- and macro-embolic risk. Therefore, the aim of the present study is to verify if the advantage of clampless OPCAB (CCAB) in minimizing neurological complications, both transient or permanent, is maintained in a cohort of patients with an extensive use of automated aorto-venous anastomoses for the revascularization of non-LAD coronary branches.

## Materials and methods

366 consecutive patients were submitted to isolated coronary bypass by a single surgeon (CB), experienced in both off and on-pump procedures, between January 2009 and December 2013. Of these patients, 223 underwent a clampless off-pump procedure.

The propensity scores (PS) were estimated by applying the logistic regression for the probability of being “treated” (surgery on-pump). The pre-operative variables included in the model were LVEF, creatinine, diabetes, previous PCI, extracardiac arteriopathy, previous CABG, carotid artery stenosis >50 and <70 %, age, sex, hypertension, smoke habit, previous MI, LMSS >50 % previous stroke, urgency, IABP. The differences between the two groups were assessed by the McNemar test for categorical variables and the Wilcoxon signed rank test for the continuos variables. Treated (on-pump) cases were individually matched to non-treated (off-pump) cases with a ratio 1:1 on the basis of the estimated PS, with a caliper equal to 20 % of the standard deviation of the logit of the PS [11].

After the propensity score adjustments, 143 pairs were selected. The characteristics of the unmatched and matched populations are shown in Table 1.

**Table 1:** Population before and after matching

Variable	Overall population (*n* = 366)			Propensity score matched (*n* = 286)		
	CCAB (*n* = 223)	CABG (*n* = 143)	*P* value	CCAB (*n* = 143)	CABG (*n* = 143)	*P* value
Age (years)	67.2 ± 9.7	67.4 ± 9.0	0.90	67.4 ± 9.4	67.4 ± 9.0	0.86
Female sex (%)	16.2	16.1	0.97	16.1	16.1	1.00
Hypertension (%)	85.1	89.5	0.23	88.1	89.5	0.71
Diabetes (%)	33.8	44.8	**0.03**	41.3	44.8	0.53
Smoke habit (%)	28.8	31.5	0.59	29.4	31.5	0.71
LVEF (%)	51.1 ± 8.5	50.8 ± 9.9	**0.03**	51.6 ± 8.9	50.8 ± 9.9	0.46
Recent MI (%)	37.4	35.0	0.64	35.7	35.0	0.90
Previous PCI (%)	18	9.8	**0.03**	9.1	9.8	0.81
Renal failure (%)^a^	5.4	2.8	0.23	2.8	2.8	1.00
Prev. CA stent (%)	2.7	2.8	0.96	2.1	2.8	0.65
CAS >50, <70 % (%)	16.7	25.9	**0.03**	22.4	25.9	0.43
Stroke/TIA (%)	10.8	10.5	0.92	11.2	10.5	0.85
LMSS (%)	37.8	31.5	0.21	30.1	31.5	0.70
PVD (%)	27.5	39.9	**0.01**	35.7	39.9	0.40
BMI (Kg/m^2^)	27.2 ± 3.7	27.5 ± 3.5	0.33	27.2 ± 3.7	27.5 ± 3.5	0.36
Urgency (%)	30.2	28.7	0.76	31.5	28.7	0.60
IABP (%)	2.3	0.7	0.25	2.1	0.7	0.16

Statistically significant *P* values are in bold

*LVEF* left ventricular ejection fraction, *MI* myocardial infarction, *PCI* percutaneous coronary intervention, *CA* carotid artery, *CAS* carotid artery stenosis, *TIA* transient ischemic accident, *LMSS* left main stem stenosis, *PVD* peripheral vascular disease, *BMI* body mass index, *IABP* intra-aortic balloon pump

^a^Renal failure: pre-operative glomerular filtration rate <50 ml/min

CCAB was performed with an all-arterial approach (*n* = 33) or by means of automated proximal anastomosis of the venous graft(s) with the Cardica PAS-Port^®^ (*n* = 110). The epicardial stabilization was obtained by means of the suction-based Medtronic Octopus and Starfish (Medtronic, Inc, Minneapolis, MN) during the first period and Maquet Acrobat and Xpose (MAQUET Cardiovascular LLC, San Jose, CA) subsequently. A pump circuit was set up in a ready-dry state. A coronary shunt was always used to maintain distal coronary flow and bloodless operative field.

The left anterior descending coronary artery was always revascularized with either a pedicled left or right ITA. In patients 65 years old or younger, both ITAs were harvested and used for the left anterior descending (right ITA) and left circumflex coronary system (left ITA). Converted patients were kept in the CCAB group since they did not receive any aortic cross- or side-clamping and were operated on by means of an assisted circulation on the beating heart.

CABG patients were operated on following a double clamping strategy, i.e., cross-clamp of the ascending aorta for the distal anastomoses followed, after myocardial reperfusion, by side-biting clamp for the proximal anastomoses. Cardioplegia was performed using antegrade, intermittent (every 20 min), warm blood cardioplegia.

The target mean arterial pressure was 90 mmHg in the CCAB group and 80 mmHg in the CABG group. If there was pre-operative demonstration of carotid vasculopathy the target of the mean arterial pressure was increased by 10 mmHg in both groups and NIRS was added to the standard monitoring system.

The choice of performing an on-pump conventional strategy was based on several reasons, such as the finding of an intramyocardial LAD, extremely enlarged left ventricle (LVEDD > 70 mm), very poor target vessels detected at first intraoperative inspection, recent episodes of electric instability, availability of the devices for off-pump procedures.

Perioperative myocardial infarction was defined as an increase of post-operative Troponin I higher than 10 ng/ml associated with a CK-MB above normal values and more than 10 % of total CK, regardless of the onset of ECG new anomalies.

We considered a post-operative respiratory failure the need of mechanical ventilation for more than 12 h due to primary (non-cardiac) pulmonary dysfunction.

Post-operative acute kidney injury (AKI) was defined as a twofold increase of pre-operative serum creatinine or oliguria necessitating continuous veno-venous hemodiafiltration.

Finally, we specified as operative mortality any death from any cause, occurring within 30 days from surgery or during hospitalization (including transferral to a cardiac rehabilitation facility) regardless of the time elapsed from the operation.

Neurologic injury was defined as non-reversible (NRNI: lethal coma or stroke, clinically diagnosed by the neurologist and afterwards instrumentally confirmed by means of cerebral MRI or CT scan) or reversible (RNI: TIA or post-operative delirium as described in the Diagnostic and Statistical Manual of Mental Disorders requiring prolonged mechanical ventilation and/or ICU stay).

The rates of post-operative events were compared with *χ*^2^ contingency tables and Fisher’s exact test (categorical variables) or Student’s *t* test for unpaired data (continuos variable), and the correspondent odds ratios were obtained with a logistic regression analysis, when possible.

## Results

The number of grafts performed per patient was 2.9 ± 0.5 in the CABG group and 2.6 ± 0.6 in the CCAB group (*p* = 0.0001). The number of grafts in the CABG and CCAB group was, respectively, 176 and 161 (*p* = 0.08) to the left anterior descending artery and its branches, 112 and 98 (*p* = 0.06) to the circumflex territory and 128 and 113 (*p* = 0.015) to the right artery territory. The duration of the surgery was 192.6 ± 29.8 min in the CABG group and 172.7 ± 38.7 min in the CCAB group (*p* = 0.0001). The overall rate of conversion was 2.8 % and was performed only from CCAB to CABG either for hemodynamic instability of the patient (2 secondary conversions) or for unsuitable target vessel (2 primary conversions, usually intramyocardial course of the artery).

Operative mortality was 2.4 % (CCAB 1.4 %, CABG 3.5 %; *p* = NS). The rate of the other early complications is reported in Table 2. In general, all the complication rates, except Troponin I dismission, favored CCAB, although the results were not, or only marginally, statistically significant. Post-operative hospital stay was 6.7 ± 4.2 days in the CCAB group, compared to 7.7 ± 7.3 days in the CABG group (*p* = NS; median: 5 and 6 days, respectively).

**Table 2:** Main post-operative outcomes

Variable	CCAB (%)	CABG (%)	*P* value	OR	95 % CL	*P* value
Mortality	1.4	3.5	0.14	0.31	0.06–1.57	0.15
TpI > 10	9.8	5.7	0.19	1.80	0.73–4.45	0.20
AKI	6.3	10.6	0.12	0.60	0.25–1.46	0.26
ARF	3.5	5.0	0.54	0.81	0.24–2.73	0.74
Bleeding	2.1	5.7	0.12	0.35	0.09–1.37	0.13
PO stay (*d*)	6.7 ± 4.2	7.7 ± 7.3	0.14	–	–	–

*OR* odds ratio for CCAB, *TpI* troponin I, *AKI* acute kidney insufficiency, *ARF* acute respiratory failure, *PO* post-operative days

### Neurological injury: matched comparisons

The global incidence of both transient and permanent neurologic injury was 5.6 % (CCAB 1.4 %, CABG 9.1 %; *χ*^2^*p* = 0.006). The incidence of NRNI was zero in CCAB group, including the 4 converted cases, and 2.8 % (4 cases: 2 lethal coma due to multiple strokes and 2 cases of unifocal stroke) in the CABG group (*χ*^2^*p* = 0.04), with a global rate of 1.4 %. As far as RNI is concerned, the difference favored CCAB versus CABG only marginally, with respective rates of 2.1 and 6.3 % (*χ*^2^*p* = 0.068; Fisher’s exact test = 0.081) and a global incidence for the entire cohort of 4.2 % (Table 3), with 5 cases of TIA (1.7 %; 1 case, or 0.7 %, in the CCAB group after a paroxysmal atrial fibrillation with spontaneous recovery and subsequent TIA, and 4 cases, or 2.8 %, in the CABG group; *p* = 0.16) and 6 cases of prolonged post-operative delirium (2.1 %; 2 cases, or 1.4 %, in the CCAB group, of which one among the 4 converted patients, and 5 cases, or 3.5 %, in the CABG group).

**Table 3:** Neurologic post-operative outcomes

Variable	CCAB (%)	CABG (%)	*P* value	OR	95 % CL	*P* value
Global NI	2.1	9.1	0.006	0.19	0.05–0.69	0.01
NRNI	0	2.8	0.04	–	–	–
RNI	2.1	6.3	0.06	0.30	0.08–1.16	0.08

*OR* odds ratio in case of CCAB, *NI* neurologic injury, *NRNI* non-reversible neurologic injury (stroke, coma), *RNI* reversible neurologic injury (TIA, delirium)

The odds ratio for NRNI could not be estimated since in the CCAB group there were no instances of the adverse event. Nonetheless, CCAB constituted a protective factor for the global rate of neurological complications and for the reversible events, as shown in Table 3.

### Neurological injury: non-matched comparisons

With respect to RNI, no differences were found inside the CCAB group between patients with totally arterial revascularization (true anaortic CCAB, *n* = 33; RNI = 3.0 %) compared to those with PAS-Port^®^-assisted proximal vein graft anastomosis (*n* = 110; RNI = 1.8 %), with *p* = 0.55.

On the other hand, the mean hospital stay was highly different in patients who suffered an RNI, compared to patients who experienced a neurologically uncomplicated post-operative course: 17.3 ± 17.8 vs. 6.5 ± 4.0 days, with *p* < 0.0001 (median value: 12 and 5 days, respectively).

## Discussion

Neurologic injury remains one of the most significant and disabling complication of coronary surgery. Coma leading to death and stroke has obviously a major impact on procedural success and costs [12]. In several meta-analysis studies, the frequency of post-operative stroke remains between 2 and 3 % in conventional CABG [13, 14] and OPCAB with side-biting clamping of the aorta failed to prevent it significantly [2, 3, 15]. On the other hand, anaortic or clampless OPCAB seems to allow a reduction in the stroke rate compared to more conventional surgical strategies [5, 7, 15]. The most common cause of permanent neurologic injury following CABG is probably embolic, and the entity and kind of aortic manipulation is considered of relevant importance in inducing this adverse event [4, 5, 16]. Therefore, avoiding any aortic cannulation and clamping should help avoiding stroke. The most intuitive way to perform an anaortic approach is the liberal use of both internal thoracic arteries, while additional grafts are anastomosed proximally on the ITA grafts. This strategy limits somehow the flow sources to the myocardium and implies a more complex and time-consuming surgery. Alternatively, either facilitating devices, like Heartstring^®^ (Maquet Cardiovascular, San Jose, CA, USA), can be used to perform hand-sewn proximal anastomoses on the aorta avoiding clamping with excellent results, or the PAS-Port^®^ system can be used to obtain automated grafting [17]. The latter device is part of our study and has a proven efficacy and safeness [18–20]. With the use of this device it is possible to achieve a clampless approach, although not truly anaortic, differentiating the flow sources and making the procedure much more expeditious (the proximal automated anastomosis requires only a few seconds to be completed).

Our data demonstrate that, while in all other major outcome variables there is no clear superiority of CCAB over CABG, although we recorded a trend towards lower complication rates for CCAB, an indisputable advantage of CCAB is evident with regard to non-reversible neurological damage even if a totally anaortic, “all arterial” strategy was achieved only in a minority of patients (23 %).

On the other hand, the less threatening complications such as TIA or prolonged post-operative delirium are reduced only partially in the CCAB group with a marginally significant difference. In more detail, the incidence of RNI was not significantly different in anaortic and non-anaortic, PAS-Port^®^-assisted cases. Therefore, we can assume that the technical differences in the CCAB group did not affect the final results and the two groups can be considered homogeneous with regards to potentially harmful aortic manipulation.

Actually, the hospital stay in patients experiencing a delayed neurological recovery is almost three times longer in the present series. Therefore, the more subtle aspects of transient neurological impairment should not be neglected in terms of patient’s discomfort and cost load.

We believe that major neurologic complications are usually macro-embolic in origin and they can be almost totally prevented by CCAB, even without a totally anaortic approach and with the help of an anastomotic connector that prevents aortic manipulation. On the other hand, it is possible that TIAs or post-operative delirium could be in part of aortic microembolic genesis, although this cannot be considered the only underlying mechanism, since reversible neurologic impairment occurs also in totally anaortic cases. Therefore, different etiological causes, such as individual reaction to anesthesia, intra-cerebral atherosclerosis, non-aortic embolism, might be responsible for the event. Consequently, RNI can be only partially avoided with such facilitating devices.

Our study has some limitations. First, an epiaortic ultrasound study was not performed in our patients, and the actual severity and distribution of proximal aorta atherosclerosis is, therefore, unknown. Anyway, since all the covariates intimately correlated with atherosclerosis were equally distributed in the study groups, one could reasonably assume that also the incidence of severely diseased ascending aorta would have been similarly distributed. Second, the sample size is relatively small and, therefore, the statistical analysis is probably underpowered to detect differences in the incidence of RNI, namely TIA and post-operative delirium.

Euroscore was calculated only for patients with relevant comorbidities and is subsequently not reported, but operative mortality was not included in the study endpoints.

The device is not cheap, its price being around 1000$. Anyway, its cost can be considered affordable based on the fact that it is able to reduce the incidence of expensive complications.

The device has several limitations in its use that have been described elsewhere [20] and cannot be considered a panacea; however, it is still a useful tool in improving the results of coronary artery bypass surgery. The liberal use of bilateral ITA grafts should be considered a milestone in coronary surgery. Nonetheless, when additional grafts are needed, a saphenous vein can be safely anastomosed to the aorta by means of the Cardica PAS-Port^®^, with a reduced risk of early embolic events and excellent long-term actual patency rates [20].

In conclusion, CCAB whether performed with all-arterial grafts or with the aid of an automatic connector that totally prevents aortic clamping appears to provide a dramatic reduction in the incidence of stroke and contributes in reducing reversible neurological damage following surgical myocardial revascularization.
